# Effect of different pH solvents on micro-hardness and surface topography of dental nano-composite: An *in vitro* analysis

**DOI:** 10.12669/pjms.314.7517

**Published:** 2015

**Authors:** Aftab Ahmed Khan, Adel Zia Siddiqui, Abdulaziz A Al-Kheraif, Ambreen Zahid, Darshan Devang Divakar

**Affiliations:** 1 Aftab Ahmed Khan, MSc, M.Bioeth, B.D.S Researcher, Dental Biomaterials Research Chair, College of Applied Medical Sciences, King Saud University, Riyadh 11433; Saudi Arabia; 2 Adel Zia Siddiqui, MSc, B.D.S Associate Professor, Dental Material Sciences, Baqai Dental College, Baqai Medical University, 51 Deh Tor, Toll Plaza, Super Highway, Gadap Road, Karachi 74600; Pakistan; 3 Abdulaziz A. Al-Kheraif, Ph.D, MSc Associate Professor and Chair, Dental Biomaterials Research Chair, College of Applied Medical Sciences, King Saud University, Riyadh 11433; Saudi Arabia; 4 Ambreen Zahid, B.D.S Lecturer, Oral Pathology, Muhammad Bin Qasim Dental College, Gulshan-e-Benazir, Razaqabad, Bin Qasim Town, Karachi; Pakistan; 5 Darshan Devang Divakar, M.D.S, B.D.S Researcher, Dental Biomaterials Research chair, College of Applied Medical Sciences, King Saud University, Riyadh 11433; Saudi Arabia

**Keywords:** Beverages, Hardness, Microscopy, Nano-filled composite, Surface degradation

## Abstract

**Objective:**

Erosion of tooth surface is attributed to recent shift in diet pattern and frequent use of beverages. The aim of this research was to evaluate the effects of different beverages on surface topography and hardness of nano-filled composite material.

**Methods:**

Sixty flat disc shaped resin composite samples were fabricated and placed in distilled water for 24 hours. After 24 hours test samples were dried and divided into 4 groups. Group A (n=15) specimens were placed in tight amber bottle comprising 25 ml of artificial saliva. Similarly Group B, C and D were stored in equal amounts of orange juice, milk and coca cola drink respectively. Samples were checked for hardness and surface changes were evaluated with scanning electron microscopy.

**Results:**

There were strong significant difference observed in samples immersed in orange juice and artificial saliva. A strong significant difference was seen between Group D and Group A. Group A and Group C showed no significant difference. The micro-hardness test showed reduced values among all samples.

**Conclusion:**

Beverages consumed daily have a negative influence on hardness and surface degradation of nano-filled dental composite. Comparatively, nano-filled composites possess higher surface area to volume ratio of their fillers particle size may lead to higher surface roughness than other resin based dental biomaterials.

## INTRODUCTION

Dental filling materials are required to possess long term durability in dynamic oral conditions. Dental restorative materials are widely used in children and adolescents, who consume different types of soft drinks leading to acidic environment.^1^ Esthetic dental biomaterials are extensively used in anterior and posterior restorations. They are available with different characteristics and shades. Four types of materials are commonly used for direct restorations: resin composites, polyacid modified resin composites, glass-ionomer cement and resin modified glass-ionomer cement.

Resin composites play key role in direct tooth colored restorations.^2^ Dentistry has been going through novel variations and advancements which tend to develop better techniques and materials for treatment of patients, who are seeking for esthetic and therapeutic treatment. Presently, resin composite show properties that are near to natural teeth.^3^ Since their improvement in the late 1950’s resin composites are widely accepted for bearing acceptable aesthetics properties and bond directly to tooth structure by preserving healthy tissues. Due to its good bonding ability it is widely accepted in preventive and conservative dentistry.^4^ Resin composite usually comprise of methacrylate polymers such as bisphenol Adiglycigyl methacrylate and tetraethylene glycol dimethacrylate. The filler particles and resin are bonded covalently by a coupling agent commonly known as 3-(trimethoxysilyl) propyl methacrylate (MPS), imparting better mechanical properties.^5^

Degradation in the oral cavity is a complex phenomenon that is related to disintegration and dissolution of restorative materials in the oral cavity. The other types of chemical/physical degradation, such as wear and erosion occur due to food, chewing and microbes. Resin composites undergo a drastic change of physical and chemical conditions in the oral cavity, including temperature changes, different masticatory stresses and chemicals from food. These factors have immense effect in vivo degradation or failure of dental composites. Exposure to various kinds of acidic solutions results in restoration failure. Researchers have claimed that exposure of resin composite to low-pH liquids can have a deleterious effect on their mechanical properties.^6^ To overcome these shortcomings of resin composite materials, a new class of dental composites have been introduced lately called nanocomposites.^7^ Nano composites are considered to have advanced mechanical properties bearing good compressive strength, and better chemical and physical properties.^8^

Hydrolysis is a chemical process that splits the covalent bonds between the polymers by adding water to ester bonds, leading in loss of resin mass and is quoted as one of the main reason of degradation of resin within the hybrid layer.^9^ Resin composites possess polymer network that is mostly inclined to hydroscopic and hydrolytic effects to varying extents, that is dependent on their chemical structure. These effects will not only affect their service life but they may also result in short term release of unreacted components as well as long term leaching of degradation products in the oral cavity.^10^ However, it was observed that leaching of different components combined with swelling and degradation of the cross linked network in resin composite and hydrolysis of filler-matrix interface finally results in decrease in mechanical properties. Degradation of resin composite may lead to formation of micro cracks via repeated sorption/desorption cycles that in turn is related with hydrolytic degradation of polymeric network.^11^

Average daily requirement to consume water is 2 to 3 liters. In developed nations more than half comes from soft drinks and fruit juices. Excessive contact of tooth with acidic drinks for a long period results in loss of hard tissues due to their low pH and highly acidic nature will eventually lead to change in surface texture.^12^ The components of acids is also responsible for the surface nano-indentations of specimens. Orange juice comprise of citric acid whereas coca cola contains phosphoric acid. In a recent study by Fan et al., revealed that the surface features of specimens immersed in orange juice were more affected than those compared with specimen aged in distilled water or cola drink.^6^ Erosion of tooth surface is attributed to recent shift in diet pattern and frequent use of beverages.^13^ Researchers report that enamel micro-hardness lowered just after one hour immersion in coca cola. Surface hardness has been used as an indirect technique to measure polymerization adequacy.^14^ In another study researchers^15^ have quoted that the type of immersion solutions and the composition of soaked materials are important factors related to dissolution of dental composite materials. On the other hand, factors such as the solubility parameter, the cross-linking nature of resin matrix, and the solvent sorption uptake may directly influence polymer degradation rate. As milk, orange juice and cola drink are consumed by every other person of different age groups so the purpose of our study was to assess the erosive potential of these commonly consumed beverages on the surface of composite material using the micro-hardness testing and scanning electron microscopy. Although limited number of studies have been previously done yet the uniqueness of this study is the surface changes observation under scanning electron microscopy.

## METHODS

The composite resin used for preparing the 60 samples of 7 mm diameter and 2 mm thickness was Te- Econom Plus (IvoclarVivadent, UK). Circular Teflon mold 7 mm in diameter and 2.0 mm in thickness was used. Mylar strip (Dentart, Polidental, Sao Paulo, Brazil) having a dimensions (10 x 120 x 0.05 mm) was placed over the top and bottom of the mold and pressed on top with a microscope slide 22 x 22 mm (BDH borosilicate glass) to form a flat surface of the sample. Light curing unit Quartz Tungsten Halogen (401™ Demetron Research Corporation, Danbury, CT, USA) with light intensity of 550 W/cm^2^ was held rigidly and placed 1.0 mm over the glass slide for 40 seconds to cure down the dental composite samples. With the help of 3M Sof-Lex disks specimens were polished to get a clinical finish. These fabricated specimens were placed in distilled water for 24 hours for post irradiation hardness. After 24 hours specimens were dried and divided into 4 groups i.e. Group A (n=15) specimens were kept in tight amber coloured bottle containing 25 ml of artificial saliva; similarly Group B (n=15), C (n=15) and D (n=15) specimens were stored in equal amount of orange juice, milk and coca cola drink respectively. Before storing the specimens at 37±3°C for 14 days in an incubator (Sanfa DNP-9052, China), the pH of all the solvents were measured by using pH meter (Model no. 98129 Hanna Instruments, USA). The immersing solutions of all the groups were changed after every 24 hours. The quantity of 25ml solution was maintained in all the groups.

After 14 days specimens were removed from the solutions and dipped into cleansing solution containing 10 ml soap and 990 ml distilled water. Later flushed under running tap water and subsequently dipped into distilled water for one minute. Fluid on the surface of the specimens were removed by using absorbing paper. Specimens were ready for micro-hardness testing.

### Preparation of Artificial Saliva

The artificial saliva used in this study was of the following composition: Sodium chloride (NaCl) 0.400 g; Potassium chloride (KCl) 0.400 g; Calcium chloride monohydrate (CaCl_2_H_2_O) 0.795 g; Sodium dihydrogen phosphate (NaH_2_PO_4_) 0.69 g; Disodium sulphide hydrate (Na_2_S x 9H_2_0) 0.005 g; Urea 1.0 g; Distilled water 1000 ml. The pH was then adjusted to 6.9 with Sodium hydroxide (NaOH) or Hydrochloric acid (HCl).

### Preparation of Experimental Solvents

Fresh orange juice, milk and coca cola soft drink was used.

### Scanning Electron Microscopy (SEM) Analysis

Surface changes were observed using the scanning electron microscopy (JEOL-JSM; 6460LV, Tokyo, Japan) at 20KV accelerating voltage.

### Vicker’s Micro-hardness Test

Micro-hardness was measured for all the 40 samples of control and testing groups. Each sample of Group A i.e. samples immersed in artificial saliva; Group B immersed in orange juice; Group C immersed in milk; and samples of Group D immersed in coca cola were employed a 50 gm load for 30 seconds dwell time. Each sample was indented for 3 times to get the mean value of the sample. In this way mean value of each group obtained. Microhardness values of the specimens were recorded using Vicker’s micro hardness tester (MMT – X7 Matsuzuwa, Japan).

### Statistical Analysis

Data was entered in Statistical Package for Social Sciences (SPSS) version 19. Descriptive analysis was executed in the form of mean ± standard deviation for surface micro-hardness. p-value of p < 0.05 were considered to be significant. Significant differences are represented by *P < 0.05. The level of significance (P) was calculated with the help of repeated measure ANOVA. For multiple comparisons, Tukey’s multiple comparison test was used.

## RESULTS

The pH of different experimental solvents used in this study are given in Table-I. The details of artificial saliva and the ingredients present in the solvents used in this study are also present in Table-I. There was a strong significance difference between Group B and Group A; a strong significance difference was also observed between Group D and Group A, and there were no statistical significant difference seen between Group A and Group C.

**Table-I:** Showing Composition and pH of various Solvents.

S. No.	Solvents	Ingredients	pH
1	Artificial Saliva	Sodium biphosphate 23%, sodium chloride 11.8%, potassium chloride 11.8%, calcium chloride 23.5%, urea 29.5%	6.9
2	Orange Juice	Carbohydrates 76%, organic acids 9.6%, free amino acids 5.4%, inorganic ions 3.2%, vitamins 2.5%, lipid 1.2%, nitrogen bases and glutathione 0.9%, flavonoids 0.8%, carotenoids 0.38%	3.5
3	Milk	Water 87%, total solids 13%, fat 4%, proteins 3.4%, lactose 4.8%, mineral 0.8%	6.0
4	Coca Cola	Carbonated water, high fructose corn syrup 70g, caramel colour, phosphoric acid, natural flavors, caffeine 53mg	2.7

The micro-hardness of the experimental material was reduced in all the groups. The micro hardness values from highest to lowest were found as follows: Group A followed by Group C then Group B and lowest in Group D. Table-II is showing mean and standard deviation value of micro-hardness of resin composite stored in different solvents.

**Table-II:** Showing Mean ± Standard deviation of Vicker’s micro-hardness values.

Groups	Immersing Mediums	n	Mean ± SD	p-value
A	Artificial Saliva	15	60.574600 ± 3.2703197	P < 0.05
B	Orange Juice	15	50.291130 ± 2.6004859	P < 0.05
C	Milk	15	58.436320 ± 2.2978890	P > 0.05
D	Coca Cola	15	39.657770 ± 1.6980882	P < 0.05

## DISCUSSION

Erosion of a resin based restorative material is a chemical process without the involvement of any micro-organisms. Erosion could be intrinsic, extrinsic or both. Consumption of easily and widely available beverages are the potential sources of resin based composite’s erosion and degradation.^12^ These beverages and drinks contain weak acids that are erosive to tooth structure. Our saliva normally has a pH of around 6.8. Whereas pure orange juice has a pH of 3.5 and that of coca cola 2.5. Milk’s pH is somewhere close to pH of saliva. Studies have shown that any drink having a pH of 5.5 or below will wear away the enamel.^16^ How long the drink stays in contact with the teeth, buffering power of the saliva, and the pH of the drink are the deciding factors in the erosion process of the tooth surfaces. Like natural tooth, effect of these acidic drinks on the restorative materials couldnot be undermined.

Sof-Lex finishing and polishing system was used because it provided the smoothest surface for composites. Before storing the specimens into experiemental solution they were stored in distilled water for 24 hours immediately after fabrication to achieve the maximum polymerization.^17^

All specimens displayed colour changes after 14 days immersion. These colour changes were noticeable to the human eye without the aid of a UV-spectrophotometer. The specimens of Group B, C and D showed marked discolouration indicating surface roughness, degradation and penetration of water molecules; leading to sorption and hydrolysis of the material. The swelling of matrix eventually leads to hydrolysis of the material.^18^ Immersion of samples in different kind of beverages containing weak acids had a profound effect on the surface deteriotion of a nano-filled composite particularly samples immersed into orange juice and cola cola drink. Matrix system present in experimental material i.e. Te econom plus is of hydrophilicnature. The higher content of organic matrix in nano-filled composites could be the reason behind higher susceptibility to water absorption and material disintegration.^14^ Since this nano-filled material had used the same polymeric matrix i.e. Bis-EMA, UDMA and TEGDMA, this result was expected and consistent with the other studies done.

**Fig. 1:**
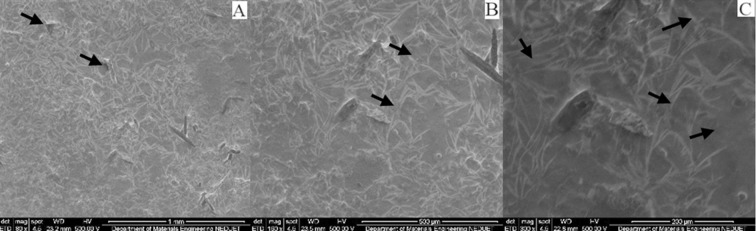
SEM micrographs of nano-filled composite specimen immersed in artificial saliva at different magnifications

**Fig. 2:**
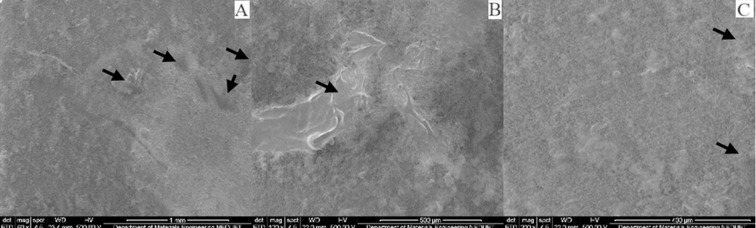
SEM micrographs of nano-filled composite specimen immersed in citric acid at different magnifications.

Irrespective of immersion medium, all specimens presented surface changes when observed under scanning electron microscopy at different magnifications. Several voids, cracks, roughness and protruding particles were observed in all specimens confirming the process of surface changes and erosion of the matrix. Although the pH of Group A specimens was 6.9 yet the artificial saliva is mainly composed of water and the effect of water uptake can degrade polymeric material.^18^ The specimens of Group B & D showed strong significant difference comparing to Group A. This was expected as the specimens of Group B & D were stored in orange juice and coca cola drink for 14 days without the buffering factor of saliva. Saliva through buffering substances keep the pH near neutral and wash away acidic environment and limit the softening of the enamel surfaces.^19^ Clinically, these beverages effects on polymeric composites may be different due to some factors such as acquired biofilm, dietary habits, and oral care products; which cannot be reproduced *in vitro*. These factors either acting alone or together may interfere with the physical and mechanical properties of the materials; influencing the durability of the polymeric resin composite. Although we daily consume acidic foods and drinks yet the composite materials show no immediate effect on their physical and mechanical properties. Excluding the samples of Group A, the samples of Group B, C and D all showed marked surface roughness and degradation (Fig. 2, 3 & 4). Samples of Group A shows surface roughness only (Fig.1). Morphological analysis of the composite material illustrates the values found in micro-hardness testing.

**Fig. 3:**
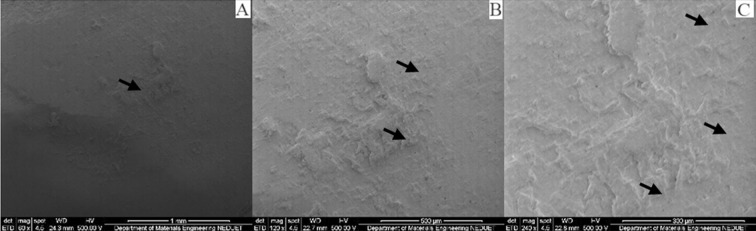
SEM micrographs of nano-filled composite specimen immersed in lactic acid atdifferent magnifications.

**Fig. 4:**
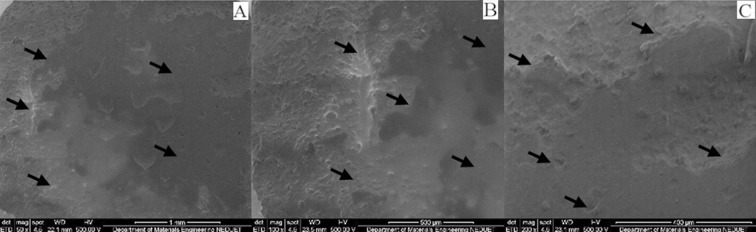
SEM micrographs of nano-filled composite specimen immersed in carbonic acid at different magnifications

The findings of this study are in accordance with earlier studies by Maganur et al.^12^ who found detrimental effects of low pH drinks on the longevity of a restorative material; significant reduction of microhardness of nano-filled composite materials when immersed in different beverages for 24 hours;^18^ ZZ significant deduction in mean values of Vicker’s hardness number due to immersion of composite specimens in cola, citric juice and wine.^20^ In a recent study conducted by Cengiz et al., it was found out that acidic medium has the tendency to soften, thereby significantly roughening dental composite biomaterial;^21^ and degradation of composite surface was also observed due to immersion in water, thermocycling and citric acid exposure.^22^

In vitro environment cannot truly depict the natural conditions of the oral cavity. In our study the disregard for the effects of saliva buffering and thermocycling is a limitation. Future research needs to test more experimental designs that could depict the clinical behavior of the restorative material *in vitro* environment. This study at least confirms the erosive potential of certain weak acids present in different juices, drinks and milk; which are a potentially damaging factor that the public should be aware of.

## CONCLUSION

Within the limitation of this study we can conclude that the weak acids present in different beverages consumed daily have a great effect on the hardness and surface roughness or degradation of the nano-filled composite material. Surface treatment, storage solution, material type and immersion time, light intensity, curing time and material thickness are important factors that influence the hardness value. Since the nano-filled composites contain greater surface area to volume ratio of their fillers particle system, this may cause them to suffer higher surface roughness or degradation as compared to other resin based materials.

## References

[1] Bajwa NK, Pathak (2014). Change in surface roughness of esthetic restorative materials after exposure to different immersion regimes in a cola drink. ISRN Dentistry 2014; Article ID 353926.

[2] Araby AME, Taher NM (2012). Effect of swimming pool water on staining susceptibility of various tooth-coloured restorative materials. KSUJDS.

[3] Tonetto MR, Neto CS, Felicio CM, Domingos PADS, Campos EAD, Andrade MFD (2012). Effect of staining agents on color change of composites. RSBO**.

[4] Schneider LFJ, Cavalcante LM, Silikas N (2010). Shrinkage Stresses Generated during Resin-Composite Applications: A Review. J Dent Biomech 2010.

[5] Koin PJ, Kilislioglu A, Zhou M, Drummong JL, Hanley L (2008). Analysis of the degradation of a model dental composite. J Dent Res**.

[6] Fan HY, Gan XQ, Liu Y, Zhu ZL, Yu HY (2014). The nanomechanical and tribological propertes of restorative dental composites after exposure in different types of media. J Nanomat 2014.

[7] Sideridou ID, Karabela MM, Vouvoudi EC (2011). Physical properties of current dental nanohybrid and nanofilled light cured resin composites. Dent Mat.

[8] Hegde MN, Hegde P, Bhandary S, Deepika K (2011). An evaluation of compressive strength of newer nanocomposite: An *in vitro* study. J Conserv Dent.

[9] Breschi L, Mazzoni A, Ruggeri A, Cadenaro M, Lenarda RD, Dorigo EDS (2008). Dental adhesion review: aging and stability of the bonded interface. Dent Mat.

[10] Bakopoulou A, Papadopoulos T, Garefis P (2009). Molecular toxicity of substances released from resin-based dental restorative materials. Int J Mol Sci.

[11] Drummond JL (2008). Degradation, fatigue and failure of resin dental composite materials. J Dent Res**.

[12] Maganur PC, Prabhakar AR, Satish V, Namineni S, Kurthukoki A (2013). Erosive effect of soft drink and fresh fruit juice on restorative materials. World J Dent.

[13] Cheng R, Yang H, Shao M, Hu T, Zhou X (2009). Dental erosion and severe tooth decay related to soft drinks: a case report and literature review. J Zhejiang Univ Sci B.

[14] Yonikoglu N, Duymus ZY, Yilmaz B (2009). Effects of different solutions on the surface hardness of composite resin materials. Dent Mat J.

[15] Munchow EA, Ferreira ACA, Machado RMM, Ramos TS, Rodrigues-Junior SA, Zanchi CH (2014). Effect of acidic solutions on the surface degradation of micro-hybrid composite resins. Braz Dent J.

[16] Seow WK, Thong KM (2005). Erosive effects of common beverages on extracted premolar teeth. Aust Dent J.

[17] Firoozmand LM, de Araujo MA (2011). Water sorption, hardness and scanning electron microscopy evaluation of dental composite resins submitted to high-risk decay model and intensive treatment with fluoride. Acta Odontol Latinoam.

[18] Tanthanuch S, Kukiattrakoon B, Siriporananon C, Omprasert N, Mettasitthikorn W, Likhitpreeda S (2014). The effect of different beverages on surface hardness of nanohybrid resin composite and giomer. J Conservat Dent.

[19] Buzalaf MAR, Hannas AR, Kato MT (2012). Saliva and dental erosion. J Appl Oral Sci.

[20] Affaf A, Gharatkar Rukshin, Irani Vijaykumar, Shiraguppi Vivek Hegde (2014). Effect of cola, orange juice, and wine on surface micro-hardness of nano-composites: An in vitro study. J Dent Orofac Res.

[21] Cengiz S, Sarac S, Özcan M (2014). Effects of simulated gastric juice on color stability, surface roughness and microhardness of laboratory-processed composites. Dent Mater J.

[22] Rinastiti M, Özcan M, Siswomihardjo W, Busscher HJ, van der Mei HC (2010). Effect of biofilm on the repair bond strengths of composites. J Dent Res.

